# Study of Response of Cotton Productivity in Southern Xinjiang to Planting Patterns and Water–Nitrogen Management

**DOI:** 10.3390/plants15040612

**Published:** 2026-02-14

**Authors:** Tingbo Lv, Menghan Bian, Fulong Chen, Conghao Chen, Maoyuan Wang

**Affiliations:** 1 College of Water Conservancy & Architectural Engineering, Shihezi University, Shihezi 832000, China; lvtingbo@126.com (T.L.); cfl103@shzu.edu.cn (F.C.); amazingcch@163.com (C.C.); 18336627918@163.com (M.W.); 2 Key Laboratory of Modern Water-Saving Irrigation of Xinjiang Production & Construction Group, Shihezi 832000, China

**Keywords:** AquaCrop, cotton productivity, drought stress, NSGA-II, planting patterns, southern Xinjiang cotton

## Abstract

To improve cotton yield and water–nitrogen productivity in arid southern Xinjiang under climate change, field experiments conducted in 2024 (for calibration) and 2025 (for validation) were conducted in Tumushuke City to evaluate planting patterns and water–nitrogen regimes. The local conventional strategy M1T3R6 (600 mm irrigation and 825 kg N ha^−1^) served as the control. Under the one-film–three-pipes–four-rows pattern (M1T3R4), three irrigation quotas (360, 450, and 540 mm) were combined with three N rates (495, 619, and 743 kg ha^−1^), and the AquaCrop model was calibrated and validated. Using 40-year (1984–2023) meteorological data and SPEI-6, hydrological years were classified into four categories: wet (Y1), normal (Y2), dry (Y3), and extreme drought (Y4). Simulations assessed cotton yield (Y), water productivity (WP), and partial factor productivity of nitrogen (PFPN) under different managements, and NSGA-II with TOPSIS was used for multi-objective optimization. AquaCrop performed well for canopy cover, soil water, evapotranspiration, and yield (R^2^ > 0.81; d > 0.85). Y, WP, and PFPN declined significantly with increasing drought severity. Compared with M1T3R6, M1T3R4 increased soil water and PFPN while reducing water and N inputs. Optimization for Y1–Y4 identified irrigation intervals of 529.9–599.1 mm and nitrogen intervals of 551.8–584.9 kg/ha, which increased yield by 8.85–21.82% while reducing irrigation by 8.33–14.15% and nitrogen by 58.6–78.1% relative to M1T3R6.

## 1. Introduction

The IPCC Sixth Assessment Report (AR6) indicates that frequent global agroecological drought events have significantly amplified hydrological process uncertainty, with climate change posing a severe threat to crop productivity under water resource constraints [1]. Against this backdrop, cotton fields in southern Xinjiang utilizing drip irrigation under plastic mulch have achieved stable yields through improved planting patterns and the “dry sowing, wet emergence” technique. However, cotton production still struggles to meet industrial demands. Moreover, the long-standing traditional model of “high irrigation quotas and excessive fertilization” leads to declining water and fertilizer productivity in farmland, while the combined effects of irrigation and nitrogen application in addressing climate change are often overlooked [2]. Therefore, there is an urgent need to enhance cotton productivity and achieve sustainable development in Southern Xinjiang through innovative planting patterns and precise water–fertilizer management.

As the link connecting the crop–soil–atmosphere system, row spacing directly influences canopy structure, evapotranspiration, and resource use efficiency. Within the row spacing range (76–102 cm) compatible with mainstream cotton pickers in Southern Xinjiang, M1T3R4, which is 76 + 10 + 76 cm (M1T3R4, 76 + 10 + 76 cm), optimizes ventilation and light penetration through wide–narrow row configurations. Compared to the traditional six-row system (M1T3R6, 10 + 66 + 10 + 66 + 10 cm), it reduces soil evaporation by 15–20% and increases light energy utilization efficiency by 12–18% [3], demonstrating its high productivity. However, reduced row spacing inevitably alters root distribution and water–nutrient demand patterns, necessitating the reconstruction of corresponding water and nitrogen management systems. Existing studies on the one-film–four-row system indicate significant threshold effects for irrigation and nitrogen application: Shi et al. [4] concluded that the medium-water–medium-nitrogen combination (280 mm and 300 kg·ha^−1^) optimizes cotton transpiration and water productivity, enhancing WP while achieving approximately 26.3% water savings; Fan et al. [5], using TOPSIS comprehensive evaluation, concluded that the high-water–high-nitrogen combination (397 mm and 300 kg·ha^−1^) best the balanced yield with water and fertilizer productivity. These divergent conclusions stem partly from the short experimental duration (only 2 years) and limited representativeness of climatic conditions and partly from differing research priorities regarding optimization objectives. Currently, no unified water–nitrogen management strategy exists for the M1T3R4 regime.

Climate change has altered annual hydrological patterns in arid regions, and cotton yield is highly sensitive to climatic fluctuations. Therefore, enhancing water–nitrogen productivity requires adapting to specific annual pattern characteristics [6]. Existing research indicates that crop growth models can overcome the short-term limitations of field trials, providing effective tools for addressing cross-annual climate risks. The AquaCrop model developed by the Food and Agriculture Organization of the United Nations (FAO) centers on a “water-driven” mechanism, offering explicit physiological representations of water stress, canopy expansion, and evapotranspiration processes. It also boasts relatively streamlined parameters and ease of calibration. A study in mulched drip-irrigated cotton fields in southern Xinjiang demonstrated that the AquaCrop model excelled in simulating canopy cover, soil moisture, and yield (R^2^ > 0.63; d > 0.86) [7]. In other arid regions, such as northern Greece [8] and South America [9], the model achieved “acceptable levels” of accuracy in simulating canopy cover and evapotranspiration. Nevertheless, AquaCrop’s default crop parameters are typically calibrated for standard row configurations under non-stressed conditions, and its performance may degrade under extreme drought (SPEI < −1.5) due to simplified stress response functions [10] or under altered planting patterns (e.g., M1T3R4 vs. M1T3R6) due to changes in canopy microclimate and root water uptake distribution. Without site-specific calibration of conservative parameters (e.g., CGC, CCx, and root expansion) under local water–nitrogen regimes, the model risks significant bias in simulating yield and water productivity for narrow–wide row cotton systems. Therefore, this study conducted two-year field calibration (2024) and validation (2025) to establish localized parameter sets before proceeding to multi-year scenario optimization. Regarding optimization strategies, previous AquaCrop-based cotton irrigation studies have primarily focused on multi-scenario simulations for single years or short-term climate trials. Existing multi-year optimizations rarely simultaneously consider indicators such as yield, water potential (WP), and partial factor productivity of nitrogen (PFPN). Junpeng et al. [11] constructed irrigation scenarios for different hydrological years in the North China Plain within the AquaCrop model. They found that crop evapotranspiration (ETa), cotton yield (Y), and water productivity (WP) exhibited quadratic relationships under high-, normal-, and low-rainfall years but did not further optimize cotton productivity. Li et al. [12] used the AquaCrop model to identify optimal irrigation schemes for cotton across precipitation years, effectively alleviating water stress. However, their approach focused solely on WP as the sole optimization target, resulting in a simplistic decision-making method. Addressing complex multi-objective decision-making has driven significant research into coupling crop growth mechanism models with intelligent optimization algorithms. The non-dominated selection genetic algorithm NSGA-II efficiently handles multi-objective nonlinear optimization problems. However, direct coupling with mechanism models incurs prohibitively high computational costs for large-scale iterations, necessitating the development of high-precision water–nitrogen–yield response functions as surrogate models [13]. Furthermore, screening Pareto frontier non-dominated solutions requires combining subjective and objective weighting, such as the entropy weight–TOPSIS method. However, current research has yet to establish an integrated framework of “AquaCrop simulation–NSGA-II optimization–TOPSIS decision-making,” particularly lacking systematic exploration of cross-seasonal water–nitrogen management for the M1T3R4 pattern.

To address these issues, this study focuses on cotton in Tumushuke City, a dry region of southern Xinjiang, establishing a “field trial–model simulation–algorithm optimization” framework. Using the local M1T3R6 cotton irrigation and nitrogen application regime as a control, a two-year field trial with two factors and three levels of water–nitrogen coupling was conducted to calibrate and validate the AquaCrop model. Using historical meteorological data from 1984 to 2023, four typical hydrological years—wet, normal, dry, and extremely dry—were selected via SPEI classification. This generated 104 water–nitrogen scenarios and established a quadratic response function for water–nitrogen–Y–WP–PFPN. Employing NSGA-II to generate Pareto solutions that maximize “Y–WP–PFPN” under each hydrological year, combined with CRITIC–subjective weighting for composite weighting, the TOPSIS method identified optimal water–nitrogen ratios balancing water conservation, nitrogen reduction, and yield enhancement to boost cotton productivity.

## 2. Results

### 2.1. Model Calibration and Validation

#### 2.1.1. Canopy Cover (CC)

**Figure 1:**
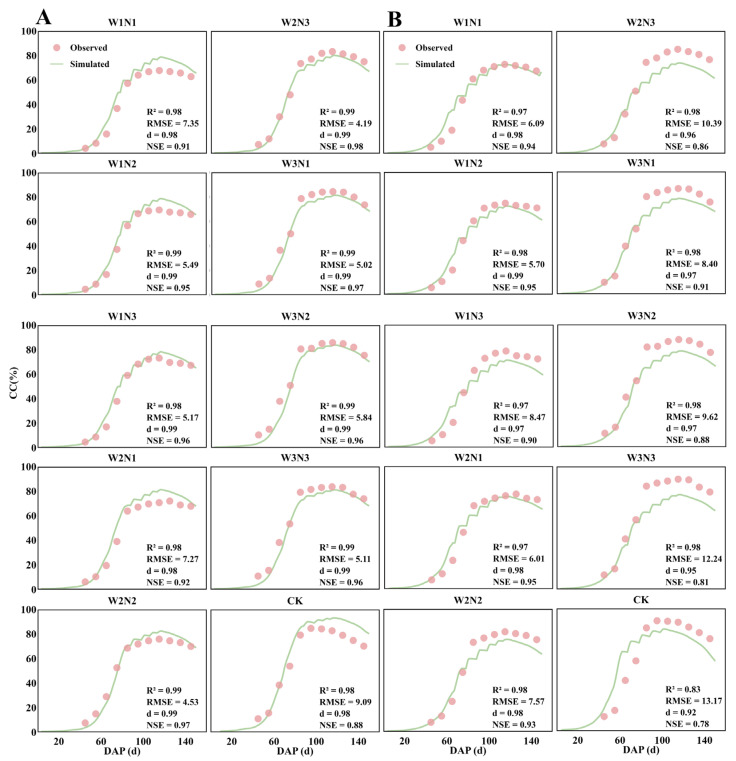
(**A**,**B**) show the observed (scatter plot) and simulated (curve) results of canopy cover CC under 10 water–nitrogen combination treatments during the cotton growing season in 2024 (calibration) and 2025 (validation), along with the values of evaluation metrics R^2^, RMSE, d, and NSE. DAP denotes days after planting.

Figure 1A and Figure 1B show the simulated and observed canopy cover (CC) values during the cotton growing seasons of 2024 and 2025, respectively. As a key variable in the AquaCrop model, CC calibration directly impacts the model’s predictive accuracy. Between 2024 and 2025, the model simulations exhibited high consistency with observed values, with R^2^ ≥ 0.83, d ≥ 0.92, RMSE ≤ 13.17%, and NSE ≥ 0.78. This indicates the model effectively simulates the trends in cotton canopy cover under various irrigation and nitrogen application combinations. Simulation results for treatments under M1T3R4 consistently outperformed the CK treatment under M1T3R6, highlighting the adaptability and advantages of this cropping pattern in model simulations. These findings confirm the reliability of the AquaCrop model for simulating cotton canopy cover and its applicability in predicting cotton growth under varying water and nitrogen treatments across different years.

#### 2.1.2. Soil Water Content (SWC)

**Figure 2:**
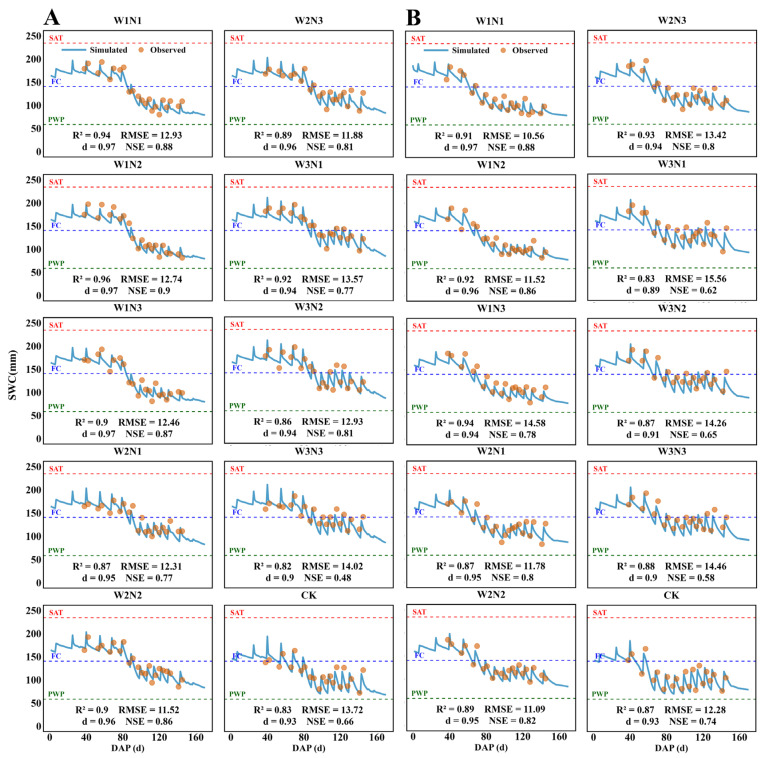
(**A**,**B**) show the observed (scatter plot) and simulated (line graph) results of soil water content (SWC) at 0–100 cm under 10 water–nitrogen combinations during the cotton growing season in 2024 (calibration) and 2025 (validation), along with the values of evaluation metrics R^2^, RMSE, d, and NSE. PWP denotes soil water volume corresponding to the wilting point moisture content, 58.61 mm; FC denotes soil water volume corresponding to field capacity, 140.67 mm; SAT denotes soil water volume corresponding to saturated capacity, 234.45 mm.

As shown in Figure 2, the AquaCrop model demonstrated excellent simulation capability between observed and simulated values during calibration and validation of soil water content (SWC) in the 0–100 cm soil profile throughout the cotton growing season in 2024 and 2025. The model responded well to different planting patterns and water–nitrogen treatments, with high R^2^, RMSE, d, and NSE values. Specifically, for 2024, these values ranged from 0.83 to 0.96, 11.52 to 14.02 mm, 0.81 to 0.97, and 0.48 to 0.9, respectively, while in 2025, these values ranged from 0.83 to 0.94, 10.56 to 15.56 mm, 0.89 to 0.97, and 0.58 to 0.9, respectively. Among all treatments, the high-water treatments (W3N1, W3N2, and W3N3) exhibited SWC values closest to the field capacity (FC) of 140.67 mm, demonstrating superior water retention capacity. Their average SWC values were 141.99 mm, 143.27 mm, and 144.83 mm, respectively, all significantly higher than the control (CK) treatment’s 111.51 mm. These results indicate that the AquaCrop model demonstrated excellent performance in simulating cotton soil moisture content for 2024 and 2025, providing a scientific basis for cotton irrigation management.

#### 2.1.3. Yield (Y) and Evapotranspiration (ET)

**Figure 3:**
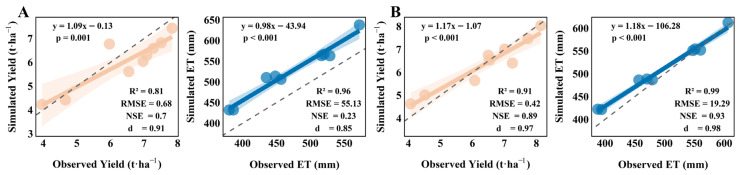
(**A**,**B**) show the correlation analysis between observed and simulated values of yield Y and evapotranspiration ET for 10 water–nitrogen treatment combinations during the cotton growing season in 2024 (calibration) and 2025 (validation), including fitted curves, fitted equations, 1:1 lines, and evaluation metrics.

Figure 3A,B show the comparison between the AquaCrop model simulations and observed data for cotton yield (Y) and evapotranspiration (ET) during the 2024 calibration phase and 2025 validation phase, respectively. The evaluation results indicate a high degree of agreement. Overall, measured Y values are uniformly distributed on both sides of the 1:1 line, indicating excellent simulation accuracy under various irrigation and nitrogen application conditions. Specifically, the M1T3R4 medium-water and high-water treatments yielded average increases of 8.05% and 21.79% over the CK treatment. Among the evaluation metrics, the 2024 R^2^ value was 0.81, RMSE was 0.68 t/ha, d was 0.91, and NSE was 0.7. In 2025, R^2^ improved to 0.99, RMSE decreased to 0.42 t/ha, d increased to 0.99, and NSE rose to 0.89, indicating enhanced simulation accuracy for the model in 2025. The ET fitting lines for both years lie slightly above the 1:1 line, indicating observed values exceed simulated values. However, the models exhibit good statistical fit with R^2^ values ranging from 0.96 to 0.99 and d values from 0.85 to 0.98. Overall, the models effectively simulate the relationship between cotton yield and ET across both years, making them a viable tool for predicting cotton growth under drip irrigation in this region.

### 2.2. Analysis of Different Hydrological Year Scenarios Based on the AquaCrop Model

**Figure 4:**
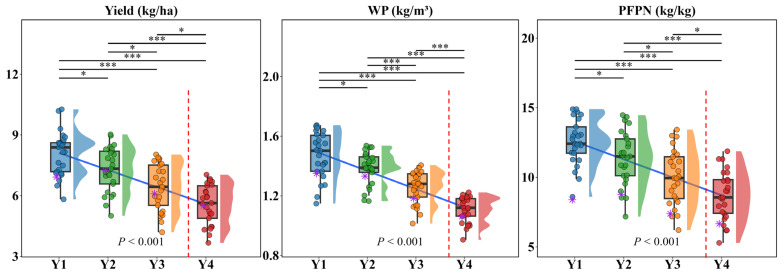
Boxplots of Y, WP, and PFPN for typical hydrological years (wet year Y1, normal year Y2, dry year Y3, and extreme drought year Y4) under different water–nitrogen combinations in the AquaCrop model. Scatter points represent the simulation scenario M1T3R4, and the purple * indicates the control scenario M1T3R6. The red dashed line represents the critical threshold (SPEI = −0.75), beyond which productivity loss accelerates significantly. Also shown are significant inter-type correlation markers (* indicates *p* < 0.05, significant; *** indicates *p* < 0.001, extremely significant), fitted curves, and *p*-values.

As shown in Figure 4, simulations of cotton yield (Y), water productivity (WP), and partial factor productivity of nitrogen (PFPN) under the calibrated AquaCrop model for Y1, Y2, Y3, Y4, and 26 water–nitrogen combinations indicate significant differences in all indicators across different annual types, with consistent response patterns to water–nitrogen management. Overall, cotton yield decreased with increasing drought severity, with Y1 exhibiting the highest average yield of 8.059 t/ha. Compared to other growth types (7.311 t/ha, 6.424 t/ha, and 5.585 t/ha), this represented increases of 10.24%, 25.46%, and 44.3%, respectively, with yield showing a stepwise decline as drought severity increased. Under identical irrigation and nitrogen application levels, growth stage emerged as the dominant factor influencing yield variation. The highest yields across all growth stages were achieved under the S25 treatment (high water + high nitrogen), reaching 10.507 t/ha, 9.427 t/ha, 8.188 t/ha, and 7.062 t/ha, respectively. The trends in WP and PFPN were highly consistent with yield, showing significant differences across all growth types and decreasing with increasing drought severity. The WP values for Y1, Y2, Y3, and Y4 ranged from 1.27 to 1.67, 1.16 to 1.54, 1.01 to 1.41 kg/m^3^, and 0.91 to 1.22 kg/m^3^ for Y1, Y2, Y3, and Y4, respectively. PFPN values ranged from 8.39 to 14.9, 7.18 to 14.47, 6.21 to 14.43, and 6.17 to 11.31 kg/kg for the same years. WP and PFPN decreased by 24.0–36.5% and 24.1–51.0%, respectively, from Y1 to Y4, following similar trends to yield. Across different cropping patterns, compared to the S26 treatment under M1T3R6, the Y, WP, and PFPN values for the medium-water–nitrogen, high-water–nitrogen, and high-water–high-nitrogen combinations under M1T3R4 averaged 22.87%, 12.35%, and 38.49% higher across all climate types, with particularly pronounced differences under drought conditions. In summary, cotton yield and water–nitrogen utilization efficiency are significantly influenced by annual climate conditions. Optimal water–nitrogen allocation and cropping patterns play a crucial role in synergistically enhancing crop productivity and resource efficiency. The M1T3R4 treatment demonstrated superior overall performance compared to M1T3R6.

To quantify the interaction between irrigation (W) and nitrogen (N) under varying drought scenarios, we examined the response surfaces derived from the 104 simulation scenarios (Appendix B). Regression analysis revealed significant W × N interaction effects on yield in Y1 and Y2 (*p* < 0.05), but the interaction term became non-significant in Y3 (*p* = 0.08) and Y4 (*p* = 0.12). Specifically, in Y1, the interaction coefficient was positive (β_3_ = 0.0004; *p* < 0.01), indicating a synergistic effect where water and nitrogen inputs enhanced each other’s productivity. However, in Y4, this synergistic effect disappeared (β_3_ = −0.0001; *p* > 0.05), suggesting that nitrogen application lost its effectiveness in sustaining yield under extreme water stress (Figure 4).

**Figure 5:**
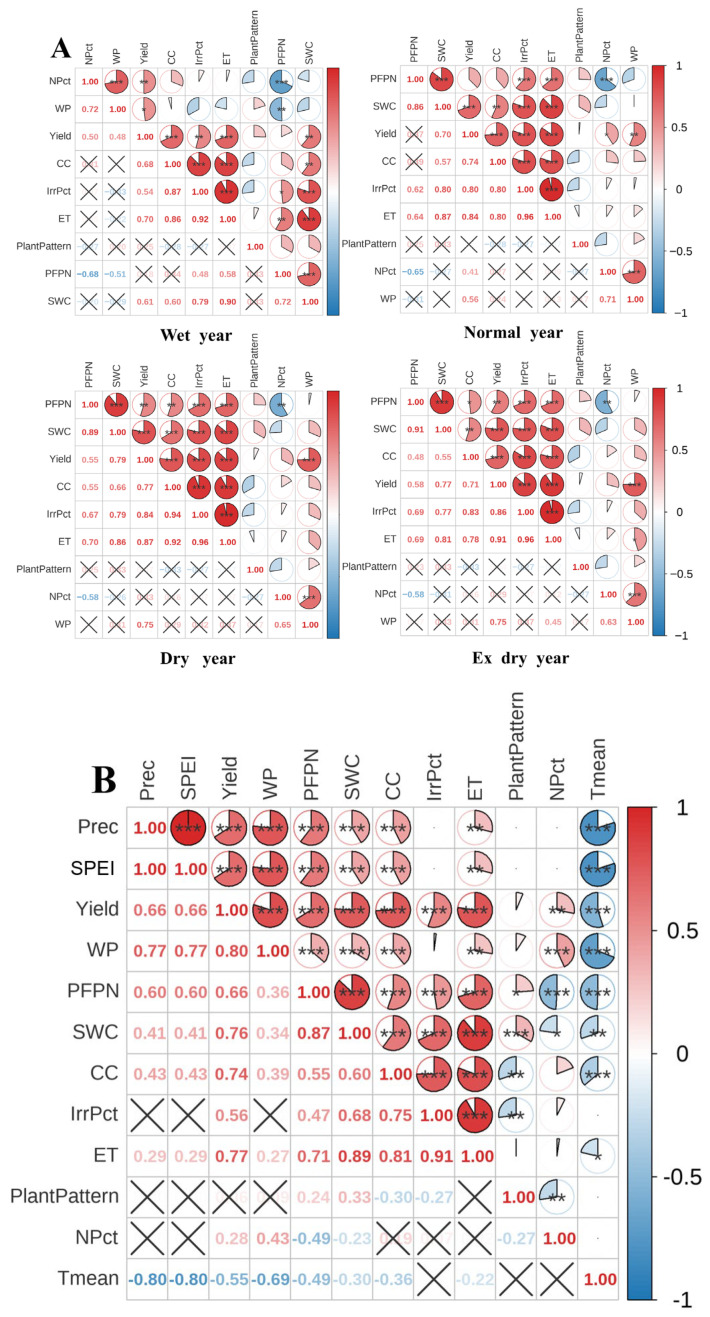
Spearman correlation analysis of variables under different water–nitrogen combinations across annual cycles in the AquaCrop model. Variables in (**A**) include Y, WP, PFPN, ET, irrigation volume, nitrogen application rate, SWC, CC, and cropping pattern (M1T3R4 denoted as 1; M1T3R6 denoted as 0); variables in (**B**) include those in (**A**) plus annual precipitation, annual temperature, and SPEI. Both figures display significant correlation markers: red indicates positive correlation and blue indicates negative correlation, with color intensity and pie chart fill representing correlation strength (* indicates *p* < 0.05, significant; ** indicates *p* < 0.01, highly significant; *** indicates *p* < 0.001, extremely significant, × indicates not significant).

Figure 5A shows the correlations between cotton yield (Y), water potential (WP), partial factor productivity of nitrogen (PFPN), and various factors. Overall, Y and WP showed a significant positive correlation (*p* < 0.05), particularly under Y3 and Y4, where it became extremely significant (*p* < 0.001). Furthermore, Y and PFPN exhibited a highly significant positive correlation only under Y3 and Y4 (*p* < 0.01). Correlation coefficients between Y and WP were 0.75 (*p* < 0.001) under Y3 and Y4, respectively; Y-PFPN correlations were only significant under drought conditions (*p* < 0.01). Figure 5B displays the correlation analysis among total variables of annual types during the cotton growth period. Results indicate that Y, WP, PFPN, and SPEI all exhibit extremely significant positive correlations, suggesting that intensified drought reduces cotton productivity. Additionally, SWC and CC showed extremely significant positive correlations with ET, further confirming the influence of the water cycle on cotton growth. Irrigation inputs exhibited extremely significant positive correlations with PFPN, while nitrogen fertilizer inputs showed extremely significant positive correlations with WP. However, nitrogen inputs also exhibited extremely significant negative correlations with PFPN and significant negative correlations with SWC, suggesting potential impacts of nitrogen application rates on water use efficiency. Regarding cropping patterns, M1T3R4 showed significant positive correlations with PFPN and SWC, while exhibiting highly significant negative correlations with water and nitrogen inputs. This indicates that M1T3R4 effectively enhances nitrogen fertilizer use efficiency and soil moisture retention capacity while reducing water and nitrogen resource consumption, with differences being more pronounced under Y3 and Y4 conditions (Table 1). These findings underscore the importance of developing differentiated irrigation and fertilization strategies under varying hydrological conditions to optimize cotton production and water–nitrogen resource use efficiency, providing a scientific basis for refining irrigation and fertilization practices.

### 2.3. Analysis of Multi-Objective Optimization Results Across Different Scenarios

**Figure 6:**
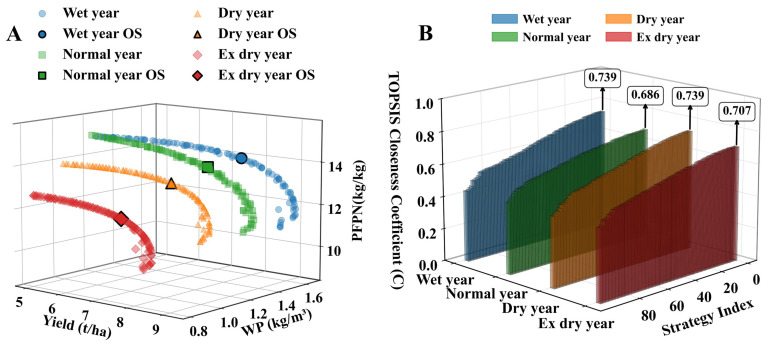
(**A**) presents the spatial distribution of the Pareto frontier under NSGA-II optimization for each cropping pattern, along with the optimal solution (OS). (**B**) displays the spatial ranking of irrigation nitrogen application decision scores (TOPSIS proximity) for each cropping pattern, showing the TOPSIS proximity C values for all Pareto solutions and the C value for the optimal solution.

As shown in Figure 6A, the scatter plot depicts the distribution of the Pareto front and optimal solutions for the three objectives—yield, WP, and PFPN—under each cropping year. The shape of the Pareto front and the trade-off relationships reveal a clear competitive trade-off among the three objectives, as evidenced by the convex Pareto front formed by all non-dominated solutions. From the perspective of different cropping seasons, the spatial distribution of the Pareto front from Y1 to Y4 shifts from the upper right to the lower left. The Pareto frontier shifted progressively from the upper-right quadrant (Y1) toward the lower-left (Y4) as drought severity increased. However, as drought stress intensifies, the scope for yield enhancement through optimization is significantly constrained, making the maintenance of water–nitrogen productivity the more critical regulatory objective. Figure 6B displays the optimal water–nitrogen management strategy selected using a combination of subjective–objective weighting and the TOPSIS method. Y1, Y2, Y3, and Y4 yield, WP, and PFPN yielded final combination weights of 0.309, 0.243, and 0.448; 0.316, 0.237, and 0.446; 0.305, 0.242, and 0.453; and 0.314, 0.246, and 0.440, respectively. This approach effectively mitigates biases inherent in single-weighting methods. The optimal solutions for each year type were determined through C-value ranking. Specifically, the optimal water–nitrogen combinations for cotton growth periods in wet years, normal years, dry years, and extreme drought years were 529.9 mm and 584.9 kg/ha, 573.7 mm and 565.8 kg/ha, 596.6 mm and 551.8 kg/ha, and 599.1 mm and 555.6 kg/ha, respectively. Detailed optimal irrigation regimes are presented in Table 1. Compared with the local S26 treatment, yield increases were 21.82%, 8.85%, 18.85%, and 15.45%, respectively. Water savings were 14.15%, 8.33%, 11.33%, and 9.14%, respectively. Nitrogen reductions were 70.66%, 58.60%, 78.10%, and 72.09%, respectively. Relative to M1T3R6 (600 mm and 825 kg/ha), the optimized M1T3R4 regimes reduced irrigation by 8.33–14.15% and N application by 58.6–78.1%, while increasing yield by 8.85–21.82%. This approach achieves significant water and nitrogen resource conservation while maintaining yield levels.

## 3. Discussion

### 3.1. Performance Evaluation of the AquaCrop Model

The determination of crop parameters plays a crucial role in the application of crop models [14]. This study, targeting a typical cotton-growing region in southern Xinjiang, achieved model localization calibration for the first time under both the M1T3R4 and traditional M1T3R6 cropping patterns. Results demonstrate that simulated values for canopy cover (CC), soil water content (SWC), evapotranspiration (ET), and yield (Y) exhibit high agreement with observed measurements (R^2^ > 0.81; d > 0.85), indicating the model effectively captures growth response differences in cotton under distinct planting patterns. Previous studies have conducted preliminary investigations into the simulation performance of the AquaCrop model for cotton in arid regions, yielding favorable evaluation results. Wang et al. [15] validated the model’s applicability to drip-irrigated cotton under plastic mulch in southern Xinjiang, where d and R^2^ values for CC and SWC approached 1 and simulated values for Y and ET showed high agreement with observed data. Song et al. [16] confirmed the model’s effective simulation of growth and yield in northern Xinjiang cotton fields, where CC’s NRMSE ≤ 20.99%, d ≥ 0.97, and R^2^ ≥ 0.91 and Y simulation RMSE = 0.389 t/hm^2^, NRMSE = 6.79%, d = 0.84, and R^2^ = 0.75. CC is a critical component of the AquaCrop model, and its precise parameterization is essential for achieving satisfactory predictions of soil moisture, evapotranspiration, and yield. In this study, measured CC values were derived from calculated cotton LAI, with corresponding parameters (CC_0_, CGC, CC_x, CDC, etc.) adjusted for different growth stages. After calibration, the simulated CC closely followed the observed trends and performed well under various water–nitrogen treatments, indicating the model’s semi-quantitative representation of nitrogen stress is accurate. The simulated SWC results under different planting patterns and water–nitrogen combinations were satisfactory, consistent with previous studies [17]. Furthermore, the simulation results exhibit significant treatment-specific differences, with the model demonstrating higher simulation accuracy for cotton yield under low-water and low-fertilizer treatments compared to high-water and high-fertilizer treatments. This aligns with the model evaluation results by Li et al. [18], indicating that calibrated parameters yield better simulations under resource-limited conditions than under fully irrigated treatments. The simulation accuracy for both yield (Y) and evapotranspiration (ET) was excellent across treatments, with R^2^ and d values of 0.86 and 0.94, and 0.98 and 0.92, respectively. ET showed slightly better fitting accuracy than Y, with simulated ET values slightly exceeding measured values. The ET overestimation reflects a structural limitation of the “ET0 × Kc” framework in simulating mulched systems. AquaCrop calculates ET as the sum of transpiration (Kc × ET0) and soil evaporation (Ke × ET0), applying a fixed mulching factor (typically 50% evaporation reduction). However, this approach underestimates the physical suppression of soil evaporation by plastic mulch, which nearly eliminates vapor exchange from the soil surface in the narrow-row configuration of M1T3R4. The model assumes residual soil evaporation contributes to ET, but in reality, the plastic barrier creates a high-humidity microclimate with minimal evaporative demand. Additionally, the standard Kc values were derived from non-mulched crops, failing to account for the altered partitioning between soil evaporation and crop transpiration under full plastic mulch coverage. This leads to systematic overestimation of ET during periods of incomplete canopy cover, when the exposed soil (between rows) should contribute minimally to total ET due to mulch suppression, but the model attributes a higher evaporation component based on unmulched reference conditions [19]. However, Ting et al. [20] suggested that the model may underestimate ET during the growing season. This discrepancy might stem from higher CC values (94–99%) in their experimental site compared to this study (67–89%). Correspondingly, the estimated 1-CC* values were typically very small, leading to simulated ET values lower than observed measurements. In summary, this study provides the first empirical confirmation of the AquaCrop model’s robust adaptability across diverse planting patterns and water–nitrogen combinations. This establishes a solid methodological foundation for subsequent meteorological scenario simulations and multi-objective optimization of outcomes, thereby enhancing cotton productivity.

### 3.2. Analysis of Simulation Scenarios for Different Hydrological Year Types

SPEI values effectively reflect the historical drought conditions in the study area. Simulation results under four typical hydrological year types classified by SPEI indicate significant differences in cotton yield (Y), water potential (WP), and plant growth and flowering period (PFPN). This demonstrates the continued necessity for differentiated management based on varying hydrological years in arid regions. Simulation results show that cotton yield decreases in a stepwise manner as drought severity increases. The average yields for Y1 to Y4 were 8.059, 7.311, 6.424, and 5.585 t/ha, respectively, representing a 30.7% reduction from Y1 to Y4 (*p* < 0.01), consistent with the threshold effect of drought stress on crop yield [21]. Further analysis revealed a significant threshold in yield response to water stress: when growing-season rainfall decreased from 89.71 mm in Y1 to 35.7 mm in Y3, yield declined by 25.39%. However, the yield reduction accelerated to 44.14% when rainfall further decreased from 15.81 mm in Y3 to 15.81 mm in Y4. This indicates that beyond a critical threshold (SPEI < −0.75), the rate of yield loss intensified under drought stress. Annual pattern differences also altered yield sensitivity to water and nitrogen inputs. In Y1, yield showed highly significant positive correlations with both irrigation volume and nitrogen application (R^2^ = 0.82 and 0.76; *p* < 0.01), with water–fertilizer synergistic effects dominating; in Y4, yield was significantly correlated only with irrigation water (R^2^ = 0.61; *p* < 0.01), while the correlation with nitrogen application weakened (R^2^ = 0.32; *p* > 0.05). The high-water–high-nitrogen treatment (600 mm and 825 kg/ha) yielded the highest output across all years, but marginal benefits decreased significantly with intensifying drought. Under Y1, Y2, Y3, and Y4, each additional 60 mm of irrigation increased yield by 6.48%, 10.00%, 12.09%, and 12.59%, respectively. This pattern has been confirmed by a large-scale meta-analysis of global dryland agriculture [22], which found that the incremental contribution of irrigation significantly declines with increasing drought severity, exhibiting clear diminishing marginal returns. The annual variation patterns of WP and PFPN differed from those of yield. WP reached 1.27–1.67 kg/m^3^ in Y1, significantly higher than the 0.91–1.22 kg/m^3^ in Y4 (*p* < 0.05), but the significance coefficients between annual types were smaller than those for yield, indicating greater stability in water productivity. PFPN exhibited more pronounced inter-seasonal variation, with a mean of 12.37 kg/kg in Y1—a 43.55% increase over Y4’s 8.61 kg/kg. This may relate to precipitation alleviating the drought’s suppression of soil water movement [23]. Regarding planting patterns, the M1T3R4 pattern significantly mitigated the negative impact of annual patterns on resource efficiency by optimizing the “row spacing–canopy–soil moisture” relationship. Compared to the control M1T3R6, M1T3R4 increased WP and PFPN by 6.06% and 36.08% on average across all annual patterns, effectively reducing water and nitrogen resource waste. The core mechanism lies in the fact that the narrow row spacing design improved the growth environment for cotton plants, reduced canopy competition, and increased water productivity (WP). Furthermore, Spearman correlation analysis indicated that the planting pattern significantly enhanced soil water content (SWC) and partial factor productivity of nitrogen (PFPN), demonstrating that M1T3R4 can improve cross-seasonal increases in soil moisture and nitrogen fertilizer partial productivity, thereby boosting cotton productivity.

### 3.3. Sustainability in Cotton Production Under Multi-Objective Decision-Making

Against the backdrop of intensifying climate change and hydrological year uncertainty, cotton productivity hinges on the dynamic equilibrium of the “yield–resource efficiency–climate adaptation” multi-objective system [24]. This study established a coupled framework integrating the AquaCrop model, the NSGA-II algorithm, and a combined subjective–objective weighted TOPSIS method. Under four hydrological year patterns, it achieved synergistic optimization of cotton yield (Y), water use efficiency (WUE), and partial factor productivity of nitrogen (PFPN), revealing the advantages of the M1T3R4 planting pattern in maintaining high productivity while reducing water and fertilizer inputs. This approach facilitates the transition of water and fertilizer management in arid cotton fields from “experience-driven” to “data-driven” practices. A quadratic response function (R^2^ > 0.85) constructed from 104 water–nitrogen scenario simulations revealed significant nonlinear responses of Y, WP, and PFPN to irrigation volume (x_1_) and nitrogen application rate (x_2_). The Pareto front generated by the NSGA-II algorithm after 5000 generations of evolution revealed a clear trade-off among the three objectives: in wet years (Y1), increasing x_1_ from 300 mm to 600 mm boosted Y by 42.7% but reduced PFPN by 28.3%; in contrast, during the extremely dry year (Y4), once x_2_ exceeded 550 kg/hm^2^, yield growth slowed while WP declined significantly. This result corroborates the theory of optimal thresholds for resource inputs. Fan et al. [25] constructed a water–nitrogen coupled production function model (R^2^ ≥ 0.80) and found a clear threshold effect in the impact of water–nitrogen management on yield: beyond the threshold, yield increases flattened, and further water–nitrogen inputs failed to yield significant gains. The optimized solution maintained target yields while reducing irrigation by 2.73% and nitrogen fertilizer inputs by 9.69%. The TOPSIS method, which coupled subjective weights (yield 0.4, WP 0.3, and PFPN 0.3) with the objective weights from CRITIC (yield 0.32, WP 0.35, and PFPN 0.33), effectively balances production demands with inherent data patterns [26]. The relative proximity C-values of optimal solutions across all cropping seasons significantly exceeded the average, indicating the method accurately identifies synergistic solutions with high yields and superior resource efficiency.

The M1T3R4 cropping pattern demonstrated significantly higher productivity advantages across all annual patterns. Compared to the traditional M1T3R6 pattern, its optimal water–nitrogen management achieved average increases of 10.74% in WP and 69.86% in PFPN, with these gains exhibiting an “N”-shaped trend as drought intensified. The annual pattern-differentiated optimization strategy proposed in this study breaks the traditional “one-size-fits-all” water–nitrogen management paradigm. Specifically: In wet years, a medium-water/high-nitrogen strategy was adopted. Natural precipitation was utilized to reduce irrigation, increasing WP to 1.54 kg/m^3^. Simultaneously, nitrogen application was moderately increased to boost PFPN to 14.32 kg/kg, achieving a yield increase to 8.43 t/ha. For normal precipitation years, a high-water–low-nitrogen strategy was adopted. Supplemental irrigation met cotton water demands while reducing WP to 1.44 kg/m^3^. Concurrently, nitrogen fertilizer was reduced to increase PFPN to 13.90 kg/kg, resulting in a slight yield decrease to 7.87 t/ha. During drought and extreme drought years, a high-water–low-nitrogen strategy was implemented. Irrigation quotas were further increased while nitrogen inputs were reduced, achieving the dual goals of saving 1.24 kg/m^3^ water and reducing nitrogen by 12.31 kg/kg. Yields remained stable at 6.35–7.25 t/ha without significant decline. The optimal irrigation and nitrogen application regime developed in this study can be directly applied to local cotton fields. It should be combined with pre-seasonal SPEI forecasts to guide farmers in adjusting water and nitrogen management [27], providing important references for agriculture in arid regions to address climate change challenges. To translate these findings into practical guidance, we propose a pre-season decision matrix linking SPEI forecasts to specific water–nitrogen prescriptions (Table 1). Prior to sowing (late March in Southern Xinjiang), farmers can obtain 6-month SPEI forecasts from regional meteorological services. Management recommendations are categorized as follows: (i) if forecasted SPEI > 0.5 (wet probability), apply the Y1 regime (529.9 mm and 584.9 kg N ha^−1^) with emphasis on nitrogen maintenance; (ii) if −0.5 < SPEI < 0.5 (normal), adopt the Y2 regime (573.7 mm and 565.8 kg N ha^−1^) balancing yield stability; (iii) if −1.5 < SPEI < −0.5 (dry), implement the Y3 regime (596.6 mm and 551.8 kg N ha^−1^) prioritizing water security; (iv) if SPEI < −1.5 (extreme drought), utilize the Y4 regime (599.1 mm and 555.6 kg N ha^−1^) maximizing water productivity while minimizing nitrogen waste. This classification system transforms complex climate projections into actionable agronomic protocols, enabling real-time adjustment of input budgets before the first irrigation event.

However, this study has limitations that warrant attention: (1) The optimization algorithm did not incorporate future meteorological data from climate change scenarios (e.g., RCP8.5). Future work could extend this approach to the watershed scale using the SWAT model, coupling machine learning algorithms to enhance parameter inversion efficiency and provide more comprehensive support for intelligent water management decision-making [28]. (2) The simplified nitrogen cycle module in the AquaCrop model may cause annual variations in PFPN bias, necessitating integration with HYDRUS model modules to refine nitrogen transport [29].

## 4. Materials and Methods

### 4.1. Overview of the Test Site

**Figure 7:**
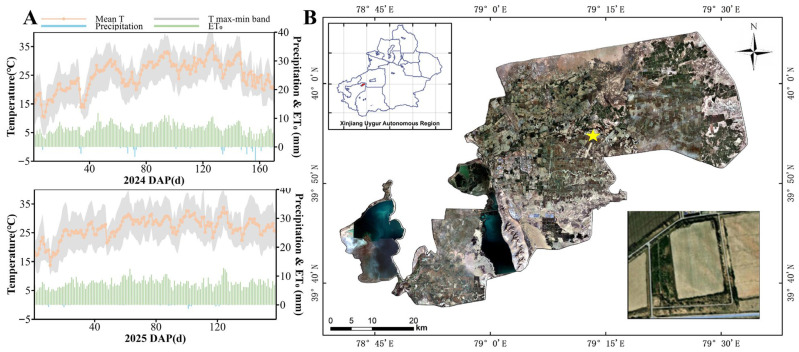
(**A**) represents the full growing season meteorological conditions for 2024–2025, while (**B**) denotes the geographical location of the experimental site. In the upper left corner, the red part of the Xinjiang map represents Tumushuke City. The yellow star indicates the location of the experimental site of this study.

The experimental site is located at the Original Seed Company of the 44th Regiment, Tumushuke City, Third Division, Xinjiang, China (79°08′05″ N, 39°55′04″ E). The site features a temperate, extremely arid desert climate at an elevation of 1096 m. The annual mean temperature is 11.6 °C, with annual precipitation of 38.3 mm. The maximum frost depth reaches 69 cm, the frost-free period spans 225 days, and groundwater lies below 3 m. Cotton meteorological data for the entire growing season in 2024 and 2025, along with the experimental site location, are shown in Figure 7A,B.

### 4.2. Experimental Design

**Figure 8:**
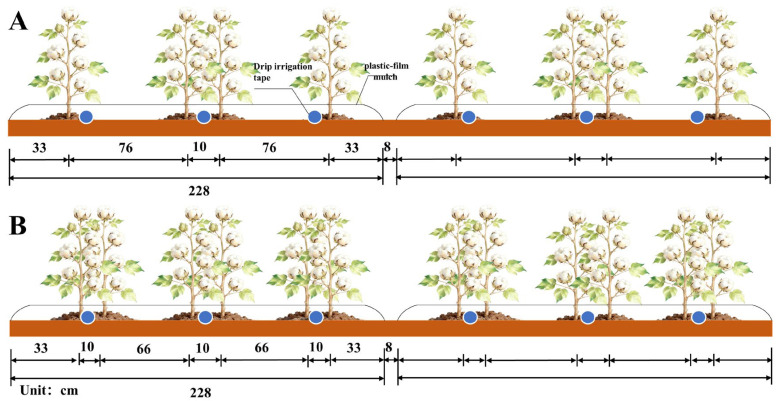
Schematic diagram of planting patterns: (**A**) represents one membrane with three tubes and four rows, while (**B**) represents one membrane with three tubes and six rows.

A randomized block design with two factors (irrigation quota [W] and nitrogen fertilizer rate [N]) at three levels was implemented from March to September in 2024 and 2025. The treatment configuration was one plastic film, three pipes, and four rows (M1T3R4, 76 cm + 10 cm + 76 cm), with three irrigation levels (W1: 360 mm, W2: 450 mm, and W3: 540 mm) and three nitrogen fertilizer application rates (N1: 495 kg/ha, N2: 619 kg/ha, and N3: 743 kg/ha). The local farmer’s irrigation and nitrogen application regime (600 mm and 825 kg/ha; one film–three pipes–six rows, M1T3R4) served as the control group (CK), resulting in 10 treatments. Each treatment was replicated three times, totaling 30 experimental plots. Each plot measured 25.08 m^2^ (11 m × 2.28 m). The theoretical planting densities for M1T3R4 and CK were 11.68 × 10^4^ and 17.52 × 10^4^ plants/hm^2^, respectively. The specific layout is shown in Figure 8.

The experimental cotton variety was “Tarahé No. 2.” The cultivation methods included mechanical mulching, perforation, and precision seeding; drone spraying of pesticides; manual harvesting; and drip irrigation using 16 mm diameter tubing with 30 cm spacing between emitters, a flow rate of 2.8 L/h, and an operating pressure of 0.1 MPa. Irrigation was conducted via drip irrigation under plastic mulch, employing dry seeding with wet emergence. Emergence irrigation was applied at 11.3 mm one day after sowing, 18 mm seven days after sowing, and 30 mm twenty-five days after sowing. Base fertilizer application comprised diammonium phosphate at 375 kg/hm^2^, urea at 225 kg/hm^2^, and potassium fulvate at 120 kg/hm^2^. For the 2024 season, sowing, topping, and harvesting occurred on 26 March, 2 July, and 25 September, respectively. For the 2025 season, these activities took place on 11 April, 6 July, and 26 September. Cotton growth stages employed a “one irrigation, one fertilizer” topdressing approach, with 11 irrigation events conducted. Fertilizers included urea (N 46%), diammonium phosphate (P 64%), and potassium fulvate (K 55%). Soil physical properties of the experimental plots are shown in Table 2, and specific irrigation designs are detailed in Table 3. All other field management practices aligned with local standards.

### 4.3. Test Items and Methods

#### 4.3.1. Soil Water Content

During the cotton growing season, soil samples were collected from each treatment film using a soil auger 2 days before and 2 days after each irrigation, repeated three times. Samples were dried and measured at depths of 0–20 cm, 20–40 cm, 40–60 cm, 60–80 cm, and 80–100 cm.

#### 4.3.2. Cotton Growth Indicators

Leaf Area Index (LAI): after final plant establishment, select three representative plants from each treatment and mark them. Measure the length and width of each leaf using a tape measure. Calculate the leaf area using the empirical coefficient formula (length × width × 0.75), then convert this value to LAI. Canopy cover (CC): this is calculated from the leaf area index using the formula

(1) $$\mathrm{CC}=1.005{\left(1-e^{-0.6\mathrm{LAI}}\right)}^{1.2},$$

#### 4.3.3. Cotton Yield

At the end of the cotton boll-opening period, measure the lint yield Y. For each treatment, delineate a representative area of 4.56 m^2^ (2.28 m × 2 m) with balanced growth within the experimental plot. Harvest the cotton and weigh it using an electronic balance (0.01 g precision). Record the number of bolls per plant, total number of plants, and total number of bolls per unit area. Determine the total boll weight, calculate the actual harvested plant density and average boll weight per plant, and convert the yield of each plot to a standard lint yield based on the treatment area.

#### 4.3.4. Water–Nitrogen Productivity

The formulas for cotton water consumption, water productivity (WP, kg/m^3^), and partial factor productivity of nitrogen (FPFN, kg/kg) are, respectively,

ET = (P + C + I) − (D + R + ∆S),(2)

WP = 0.1Y/ET(3)

PFPN = Y/N(4)

In the equation: ET represents crop water consumption, mm; P denotes precipitation, mm; C signifies groundwater recharge, mm; I indicates total irrigation water supply over the entire growing season, mm; D represents deep percolation, mm; R denotes surface runoff, mm; and ∆S is the change in soil water storage, mm. (In this experimental area, groundwater is deeply buried; surface evaporation is intense; and C, D, and R are all zero). N represents nitrogen fertilizer application rate for cotton, kg/hm^2^.

### 4.4. AquaCrop Model Overview

#### 4.4.1. Model Principle

The AquaCrop model is a water-driven model developed by the FAO that simulates crop growth and predicts yields based on meteorological data, CO_2_ concentrations, crop growth parameters, and field management practices. It primarily simulates aboveground biomass and yield by regulating soil moisture. Compared to traditional yield–moisture response equations, this model incorporates the following improvements and optimizations [30]: it employs canopy cover (CC) instead of the traditional leaf area index (LAI), utilizes normalized water productivity (WP*) to convert crop transpiration into aboveground biomass (B), and calculates grain yield (Y, t/ha) based on final aboveground biomass and the harvest index (HI, %).

#### 4.4.2. Establishment of the Model Database

Meteorological Module: data obtained from the Tumushuke Meteorological Bureau primarily includes daily average temperature (Tmean, °C), maximum temperature (Tmax, °C), minimum temperature (Tmin, °C), precipitation (Rain, mm), relative humidity (RHmean, %), wind speed (u(x), m/s), and solar radiation (Rs, MJ/m^2^·day). Parameters are adjusted using the FAO-recommended Penman–Monteith formula, incorporating meteorological station coordinates (elevation and latitude) to enable the model to autonomously calculate reference crop evapotranspiration (ET0).

Crop Module: this module configures different cropping patterns: M1T3R4 and M1T3R6. Conservative parameters (unchanged by geographic location, crop planting time, or field management practices) can directly adopt calibration values recommended in the model manual. Non-conservative parameters (e.g., planting density, canopy cover CC, canopy growth coefficient CGC, and decay coefficient CDC) require localization adjustments. Following the methodology of Li et al. [18], the calibration employs the “OTA trial-and-error method” combined with field measurements. The OTA (One-Time Adjustment) method involved iterative parameter tuning against observed CC trajectories [18]. For CDC calibration: starting from FAO defaults, CDC was adjusted in 0.02%/GDD increments (range: 0.15–0.35%/GDD) by comparing simulated vs. observed CC at four senescence stages (DAP 100, 115, 130, and 145). The calibration converged when NSE > 0.75 and RMSE < 8% for the decline phase (DAP 100—harvest), yielding 0.25%/GDD for M1T3R4 (vs. 0.31 for M1T3R6), reflecting faster canopy decline in narrow-row configurations. This procedure was repeated for CC_0_ (±0.05% adjustment), CGC (±0.2%/d), and KcTR (±0.05) until global metrics (R^2^ > 0.80 and NSE > 0.70) were satisfied across all 10 treatments. The finalized parameters are detailed in Table 4.

Soil Module: primarily includes soil texture, field capacity at various depths, saturation water content, permanent wilting point, and bulk density. Soil physical properties for the experimental site are listed in Table 1.

Field Module: covers irrigation schedules and field management practices, mainly including soil fertility level (moderate fertility) and agronomic measures (plastic mulching, no surface runoff, and no weed growth); crop irrigation regime (irrigation frequency, irrigation dates, and irrigation rate; see Table 2 for details); and irrigation method (drip irrigation, with 30% of the soil surface kept moist).

#### 4.4.3. Model Evaluation

The model was calibrated and validated using experimental data from 2024 and 2025 for canopy cover (CC), soil water content (SWC), yield (Y), and evapotranspiration (ET), respectively. Performance was assessed using root mean square error (RMSE), Nash efficiency coefficient (NSE), synergy index (d), and coefficient of determination (R^2^). RMSE quantifies the absolute deviation between observed and simulated values, providing an intuitive measure of numerical discrepancy; NSE comprehensively evaluates the model’s ability to explain observed variance and control error, balancing correlation and systematic bias; R^2^ focuses on the proportion of linear variation in measured values explained by simulated values, highlighting trend fit; and d characterizes the overall consistency between the two sets of values in terms of trend and magnitude, enhancing the comprehensive assessment of agreement. A lower RMSE, higher NSE, and a closer value to 1 indicate superior simulation performance. Most studies suggest that models with R^2^ > 0.8 are suitable for crop simulation. These thresholds were selected based on established criteria in agricultural systems modeling: R^2^ > 0.80 indicates acceptable correlation; NSE > 0.50 (preferably > 0.75) denotes satisfactory predictive skill; d > 0.85 confirms good agreement; and RMSE values lower than 15% for CC, 20 mm for SWC, and 0.8 t/ha for yield are considered acceptable for field-scale crop model validation.

#### 4.4.4. Model Scenario Setup

Based on historical meteorological data for the cotton growing season from 1984 to 2023, annual patterns were classified using the Standardized Precipitation Evapotranspiration Index (SPEI). While time scales of 1, 3, 6, or 12 months are generally applicable, this study employed a 6-month scale covering April to September annually. This approach comprehensively considers the water balance between precipitation and potential evapotranspiration, precisely aligning with the climatic characteristics of arid regions. Specific historical meteorological data is presented in Appendix A. The year with the smallest deviation from the median SPEI value for each climate type was selected as the representative year: 2017 as a wet year (SPEI = 0.91), 2014 as a normal year (SPEI = −0.06), 1999 as a drought year (SPEI = −0.75), and 1985 as an extreme drought year (SPEI = −1.73). Under the M1T3R4 cropping pattern, there are five irrigation regimes (60%, 70%, 80%, 90%, and 100% of local irrigation) (600 mm and 825 kg/hm^2^), with the M1T3R6 local irrigation regime serving as the control. This configuration yielded 104 scenarios (5 × 5 × 4 + 1 × 4), detailed in Appendix B. Furthermore, the AquaCrop model does not account for soil nutrient cycling and balance. Instead, it provides a semi-quantitative method to determine the impact of soil fertility stress on crops, manifested as reductions in Brel, CGC, CCx, average CC, and WP*. Referencing previous experimental results [31,32], the specific scenario simulations are detailed in Table 5.

### 4.5. Multi-Objective Optimization Process for Water and Nitrogen Management

**Figure 9:**
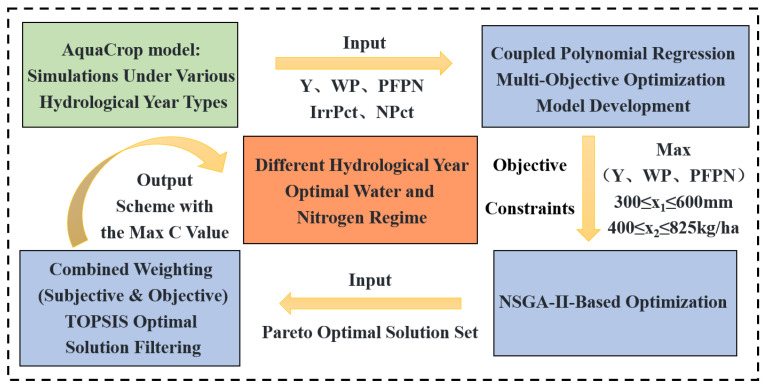
Optimization flowchart for optimal water and nitrogen management regime of M1T3R4 cotton in Southern Xinjiang.

As shown in Figure 9, this study constructed a three-objective optimization model for cotton yield–WP–PFPN based on simulation results from 104 water–nitrogen scenarios using the AquaCrop model. The NSGA-II algorithm and a combined subjective–objective weighting–TOPSIS method were employed to select recommended water–nitrogen regimes for each hydrological year type. First, a quadratic polynomial regression was employed to construct the objective function, with specific formulas detailed in Appendix C. Subsequently, a multi-objective optimization model was established with yield, WP, and PFPN maximization as optimization targets and irrigation volume (x_1_) and nitrogen application rate (x_2_) as constraints, as shown in Equation (5). Subsequently, the NSGA-II algorithm was employed to identify Pareto frontier solutions, with agricultural water–nitrogen optimization parameters configured as follows [33]: population size: 100; crossover operator: simulated binary crossover (SBX; crossover probability 0.9 and distribution exponent 20); mutation operator: polynomial mutation (PM; mutation probability 0.1 and distribution exponent 20); termination condition: 5000 generations. Targeting increased cotton yield, water savings, and reduced nitrogen use in arid regions, subjective weights for yield, WP, and PFPN were set at 0.4, 0.3, and 0.3, respectively. Objective weighting was performed using CRITIC, and subjective and objective weights were fused via the multiplicative synthesis normalization method. Finally, the TOPSIS (Technique for Order Preference by Similarity to Ideal Solution) method was applied to calculate the proximity of each scheme to the positive ideal solution (optimal target combination) and negative ideal solution (worst target combination), enabling scheme ranking and selection. The optimal solution was screened based on the relative proximity coefficient C.

(5) $$\left\{\begin{array}{ll} max & \;\;\;\;{\;\;\;\;\;\;\;\;f}_{1}\left(x_{1},x_{2}\right)=Y\left(x_{1},x_{2}\right)\\ max & \;\;\;\;\;\;\;\;\;f_{2}\left(x_{1},x_{2}\right)=WP\left(x_{1},x_{2}\right)\\ max & \;\;\;\;\;\;f_{3}\left(x_{1},x_{2}\right)=PFPN\left(x_{1},x_{2}\right)\\ s.t. & 300\leq x_{1}\leq 600,\;400\leq x_{2}\leq 825 \end{array}\right.$$

In the equation, *s.t.* denotes the constraints.

### 4.6. Data Processing and Analysis

Data organization was performed using Excel 365 software, while statistical analysis and visualization were completed using the R programming language: ① correlation analysis: stratified Spearman correlation analysis by year and type, controlling for false positives in multiple comparisons; ② nonparametric testing: Wilcoxon rank-sum test for pairwise comparisons and significance labeling of indicators across different years and types; ③ regression analysis: linear regression-fitted trends of indicators with drought severity and output model significance. Graphing additionally utilized Origin2022 software; the NSGA-II algorithm, combined subjective–objective weighting, and TOPSIS method screening were executed using Python 3.14.

## 5. Conclusions

This study calibrated and validated the AquaCrop model using field trial data from 2024 to 2025 in typical drip-irrigated cotton fields of southern Xinjiang’s arid region. It constructed scenarios of different cropping patterns and water–nitrogen combinations under varying hydrological years and analyzed the effects of different treatments on cotton yield (Y), WP, and PFPN under different planting patterns and water–nitrogen combinations. The “AquaCrop–NSGA-II–TOPSIS” approach was employed for multi-objective scenario optimization, proposing cotton water–nitrogen management strategies tailored to different hydrological years to enhance cotton productivity. Key findings are as follows:

(1) The AquaCrop model demonstrates good applicability in drip-irrigated cotton fields of southern Xinjiang. After two years of data calibration, the model demonstrated high simulation accuracy for CC, SWC, B, and Y (R^2^ > 0.81; d > 0.85). It effectively simulated cotton growth, development, and yield formation under various irrigation and nitrogen application treatments in a single-film–three-pipe–four-row configuration, providing a reliable tool for historical scenario analysis and multi-objective optimization.

(2) Different hydrological years significantly impacted cotton productivity and water–nitrogen use efficiency in southern Xinjiang. As drought severity increased, cotton yield exhibited a stepwise decline overall, with extreme drought years showing up to 44.14% lower yields compared to relatively moist years. The M1T3R4 treatment outperformed M1T3R6 across all rainfall patterns, increasing WP and PFPN by approximately 6.06% and 36.08% on average. The improvement was particularly pronounced during drought and extreme drought years, demonstrating the high adaptability of wide–narrow row spacing combined with drip irrigation under plastic mulch to drought stress, as well as its potential for efficient utilization of soil moisture and nitrogen fertilizers.

(3) Multi-objective optimization results indicate that M1T3R4 achieves increased cotton yields with reduced water and nitrogen inputs, demonstrating high productivity advantages. This study established a year-type differentiated water–nitrogen management system balancing yield increase, water conservation, and nitrogen reduction. The optimal irrigation range for each year type was 529.9–599.1 mm, with nitrogen fertilizer application ranging from 551.8 to 584.9 kg/ha. Compared to M1T3R6, this approach increased yields by 8.85–21.82%, while reducing water consumption by 8.33–14.15% and nitrogen application by 58.60–78.10%. This provides technical support for cotton production in arid southern Xinjiang to mitigate climate change impacts.

## Figures and Tables

**Table 1:** Optimal water–nitrogen combination irrigation regimes for each crop year.

Irrigation System	Hydrological Year Type	Cotton Growth Stages								
		Seedling Stage		Bud Stage		Flowering and Boll-Setting Stage		Boll-Opening Stage		Relative Efficiency Gain/%
		Number of Times	Total Volume	Number of Times	Total Volume	Number of Times	Total Volume	Number of Times	Total Volume	
Watering volume/mm	Wet year	2	85.6	2	85.6	6	256.7	1	42.8	14.15
	Normal year	2	93.5	2	93.5	6	280.6	1	46.8	8.33
	Dry year	2	97.7	2	97.7	6	293.1	1	48.8	11.33
	Ext. dry year	2	98.1	2	98.1	6	294.4	1	49.1	9.14
Nitrogen fertilizer application rate/kg/ha	Wet year	2	65.4	2	65.4	6	196.3	1	32.7	70.66
	Normal year	2	62.0	2	62.0	6	185.9	1	31.0	58.60
	Dry year	2	59.4	2	59.4	6	178.3	1	29.7	78.10
	Ext. dry year	2	60.1	2	60.1	6	180.3	1	30.1	72.09

**Table 2:** Physical properties of soil in experimental area.

Soil Depth (cm)	Soil Bulk Density (g/cm^3^)	Field Capacity (cm^3^/cm^3^)	Saturated Water Content (cm^3^/cm^3^)	Clay Particles (%)	Powder Granules (%)	Sand Grain (%)	Wilting Coefficient (cm^3^/cm^3^)
0–20	1.45	0.21	0.28	17.80	30.85	51.35	0.07
20–40	1.46	0.22	0.31	20.68	27.23	52.09	0.08
40–60	1.48	0.24	0.32	20.53	27.35	52.12	0.10
60–80	1.51	0.24	0.29	19.52	25.93	54.55	0.09
80–100	1.51	0.22	0.28	19.62	24.21	56.17	0.08

**Table 3:** Irrigation system in cotton growing period.

Cotton Growth Period	Irrigation Date /Month. Day		Irrigation Quota /mm			Nitrogen Fertilizer /(kg/ha)				
	2024	2025	W1	W2	W3	CK	N1	N2	N3	CK
Emergence stage	3.27	4.13	11.3	11.3	11.3	11.3	0.0	0.0	0.0	0.0
	4.02	4.22	18	18.0	18.0	18.0	0.0	0.0	0.0	0.0
	4.20	5.10	30.0	30.0	30.0	30.0	0.0	0.0	0.0	0.0
Seedling stage	5.05	5.20	27.3	35.5	43.7	49.2	27.0	39.4	51.8	60.0
	5.20	6.01	27.3	35.5	43.7	49.2	27.0	39.4	51.8	60.0
Bud stage	6.02	6.12	27.3	35.5	43.7	49.2	27.0	39.4	51.8	60.0
	6.13	6.22	27.3	35.5	43.7	49.2	27.0	39.4	51.8	60.0
Flowering and boll-setting stage	6.23	6.30	27.3	35.5	43.7	49.2	27.0	39.4	51.8	60.0
	7.03	7.08	27.3	35.5	43.7	49.2	27.0	39.4	51.8	60.0
	7.11	7.15	27.3	35.5	43.7	49.2	27.0	39.4	51.8	60.0
	7.18	7.23	27.3	35.5	43.7	49.2	27.0	39.4	51.8	60.0
	7.26	7.30	27.3	35.5	43.7	49.2	27.0	39.4	51.8	60.0
Batting	8.03	8.06	27.3	35.5	43.7	49.2	27.0	39.4	51.8	60.0
	8.15	8.16	27.3	35.5	43.7	49.2	0.0	0.0	0.0	0.0
Total	-	-	360.0	450.0	540.0	600.0	495	619	743	825.0

**Table 4:** Key parameters of the calibrated AquaCrop model.

Symbol	Project	M1T3R4 Value	M1T3R6 Value	Unit	Remarks
CC0	Initial canopy cover	0.57	0.85	%	Measured
CCx	Maximum canopy cover	94	98	%	Measured
CGC	Canopy growth coefficient	7	7.7	%/d	Calibrated
CDC	Canopy decay coefficient	0.25	0.31	%/GDD	Calibrated
Zm	Minimum effective root depth	0.2	0.2	m	Measured
Zx	Maximum effective root depth	0.8	0.7	m	Measured
KcTR	Crop transpiration coefficient	1.15	1.2	-	Calibrated
Rexshp	Root growth rate	0.7	0.7	cm/d	Recommended
fshape, z	Shape factor of root zone expansion	1.5	1.5	-	Recommended
WP*	Moisture productivity standardized by ET_o_ and CO_2_	21	21	g/m^2^	Recommended
HI0	Reference yield index	40	37	%	Measured
KCTr, x	Crop coefficient at full canopy integrity	0.6	1.1	-	Calibrated
fexp, w	Water stress morphological factor canopy expansion coefficient	3.5	3.5	-	Recommended
Pexpupper	Upper limit of water stress effect on canopy growth	0.55	0.7	-	Calibrated
Pexplower	Lower limit of water stress effect on canopy growth	0.15	0.2	-	Calibrated
Psto	Percentage of TAW at stomatal closure initiation	0.4	0.4	-	Recommended
fshape, sto	Water stress morphological factor stomatal closure coefficient	0.55	0.55	-	Recommended

**Table 5:** Fertilizer stress parameter settings for different nitrogen application treatments in the model.

Fertilizer Application Rate/% of Local Rate	Calibration Input Parameters			Calibration Result Parameters			
	Relative Biomass Brel/%	Maximum Canopy Cover CCx/%	Canopy Attenuation Degree	Average Canopy Reduction /%/Day	Crown Expansion Rate Reduction (%)	Maximum Canopy Cover CCx Reduction (%)	Crop Water Productivity WP* Reduction (%)
100	Not considered						
90	95	94	Small	0	0	0	10
80	90	89	Small	0.03	3	5	17
75	80	86	Small	0.13	7	9	26
70	65	75	Small	0.25	10	20	37
60	55	61	Small	0.39	17	35	43

**Figure A1:**
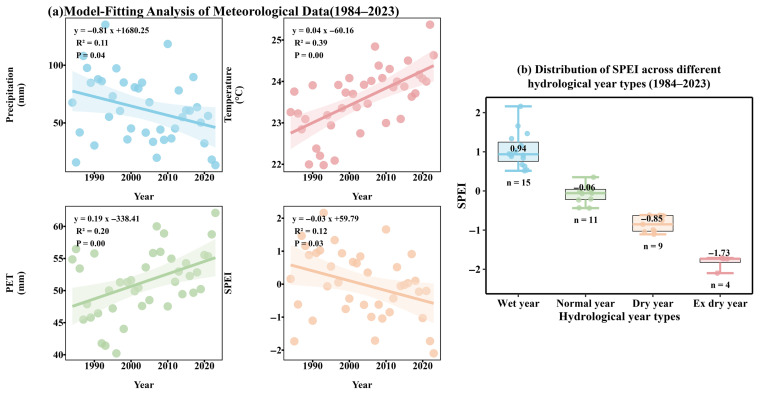
(**a**) shows the annual precipitation (mm), mean annual temperature (°C), evapotranspiration (PET), and SPEI distribution for cotton growing seasons in Tumushuke City from 1984 to 2023. In panel (**b**), the specific classification of four typical hydrological years is presented. These cotton hydrological years are classified into four drought severity levels according to SPEI values: wet years (>0.5), normal years (−0.5 to 0.5), dry years (−1.5 to −0.5), and extremely dry years (<−1.5).

## Data Availability

All data supporting this study are included in the article.

## Appendix B

**Table A1:** Different water–nitrogen combination settings under four distinct hydrological years in model simulations. N is nitrogen fertilizer application rate, I is irrigation quota, S1–S25 are M1T3R4, and S26 is M1T3R6.

Scenario	Hydrological Year Type	N /kg/hm^2^	I /mm	Scenario	Hydrological Year Type	N /kg/hm^2^	I /mm
Y1S1	Wet year	495	360	Y2S1	Normal year	495	360
Y1S2	Wet year	495	420	Y2S2	Normal year	495	420
Y1S3	Wet year	495	480	Y2S3	Normal year	495	480
Y1S4	Wet year	495	540	Y2S4	Normal year	495	540
Y1S5	Wet year	495	600	Y2S5	Normal year	495	600
Y1S6	Wet year	578	360	Y2S6	Normal year	578	360
Y1S7	Wet year	578	420	Y2S7	Normal year	578	420
Y1S8	Wet year	578	480	Y2S8	Normal year	578	480
Y1S9	Wet year	578	540	Y2S9	Normal year	578	540
Y1S10	Wet year	578	600	Y2S10	Normal year	578	600
Y1S11	Wet year	660	360	Y2S11	Normal year	660	360
Y1S12	Wet year	660	420	Y2S12	Normal year	660	420
Y1S13	Wet year	660	480	Y2S13	Normal year	660	480
Y1S14	Wet year	660	540	Y2S14	Normal year	660	540
Y1S15	Wet year	660	600	Y2S15	Normal year	660	600
Y1S16	Wet year	723	360	Y2S16	Normal year	723	360
Y1S17	Wet year	723	420	Y2S17	Normal year	723	420
Y1S18	Wet year	723	480	Y2S18	Normal year	723	480
Y1S19	Wet year	723	540	Y2S19	Normal year	723	540
Y1S20	Wet year	723	600	Y2S20	Normal year	723	600
Y1S21	Wet year	825	360	Y2S21	Normal year	825	360
Y1S22	Wet year	825	420	Y2S22	Normal year	825	420
Y1S23	Wet year	825	480	Y2S23	Normal year	825	480
Y1S24	Wet year	825	540	Y2S24	Normal year	825	540
Y1S25	Wet year	825	600	Y2S25	Normal year	825	600
Y1S26	Wet year	825	600	Y2S26	Normal year	825	600
Y3S1	Dry year	495	360	Y4S1	Extremely dry year	495	360
Y3S2	Dry year	495	420	Y4S2	Extremely dry year	495	420
Y3S3	Dry year	495	480	Y4S3	Extremely dry year	495	480
Y3S4	Dry year	495	540	Y4S4	Extremely dry year	495	540
Y3S5	Dry year	495	600	Y4S5	Extremely dry year	495	600
Y3S6	Dry year	578	360	Y4S6	Extremely dry year	578	360
Y3S7	Dry year	578	420	Y4S7	Extremely dry year	578	420
Y3S8	Dry year	578	480	Y4S8	Extremely dry year	578	480
Y3S9	Dry year	578	540	Y4S9	Extremely dry year	578	540
Y3S10	Dry year	578	600	Y4S10	Extremely dry year	578	600
Y3S11	Dry year	660	360	Y4S11	Extremely dry year	660	360
Y3S12	Dry year	660	420	Y4S12	Extremely dry year	660	420
Y3S13	Dry year	660	480	Y4S13	Extremely dry year	660	480
Y3S14	Dry year	660	540	Y4S14	Extremely dry year	660	540
Y3S15	Dry year	660	600	Y4S15	Extremely dry year	660	600
Y3S16	Dry year	723	360	Y4S16	Extremely dry year	723	360
Y3S17	Dry year	723	420	Y4S17	Extremely dry year	723	420
Y3S18	Dry year	723	480	Y4S18	Extremely dry year	723	480
Y3S19	Dry year	723	540	Y4S19	Extremely dry year	723	540
Y3S20	Dry year	723	600	Y4S20	Extremely dry year	723	600
Y3S21	Dry year	825	360	Y4S21	Extremely dry year	825	360
Y3S22	Dry year	825	420	Y4S22	Extremely dry year	825	420
Y3S23	Dry year	825	480	Y4S23	Extremely dry year	825	480
Y3S24	Dry year	825	540	Y4S24	Extremely dry year	825	540
Y3S25	Dry year	825	600	Y4S25	Extremely dry year	825	600
Y3S26	Dry year	825	600	Y4S26	Extremely dry year	825	600

## Appendix C

**Table A2:** Objective function for bivariate quadratic polynomial regression.

Year Type	Target Indicators	Quadratic Regression Equation	R^2^	RMSE
Y1	yield	−21.2414 + 0.0660 × x_1_ + 0.0331 × x_2_ + −0.0001 × x_1_^2^	0.74	0.55
Y1	wp	−1.4779 + 0.0048 × x_1_ + 0.0051 × x_2_	0.84	0.06
Y1	pfpn	−17.4365 + 0.1070 × x_1_ + 0.0185 × x_2_ + −0.0001 × x_1_^2^	0.85	0.69
Y2	yield	−19.1892 + 0.0573 × x_1_ + 0.0296 × x_2_	0.91	0.32
Y2	wp	−1.7905 + 0.0057 × x_1_ + 0.0050 × x_2_	0.87	0.04
Y2	pfpn	−17.5426 + 0.1003 × x_1_ + 0.0165 × x_2_ + −0.0001 × x_1_^2^	0.96	0.40
Y3	yield	−21.6472 + 0.0509 × x_1_ + 0.0389 × x_2_	0.91	0.33
Y3	wp	−2.5087 + 0.0057 × x_1_ + 0.0066 × x_2_	0.90	0.03
Y3	pfpn	−23.3574 + 0.0914 × x_1_ + 0.0340 × x_2_ + −0.0001 × x_1_^2^	0.96	0.40
Y4	yield	−16.7853 + 0.0421 × x_1_ + 0.0293 × x_2_	0.93	0.25
Y4	wp	−1.8869 + 0.0048 × x_1_ + 0.0051 × x_2_	0.90	0.03
Y4	pfpn	−17.2082 + 0.0773 × x_1_ + 0.0218 × x_2_	0.97	0.30
